# Impact of age on the prognosis of patients with ventricular tachyarrhythmias and aborted cardiac arrest

**DOI:** 10.1007/s00391-022-02131-6

**Published:** 2022-12-08

**Authors:** Kathrin Weidner, Tobias Schupp, Jonas Rusnak, Ibrahim El-Battrawy, Uzair Ansari, Jorge Hoppner, Julian Mueller, Maximilian Kittel, Gabriel Taton, Linda Reiser, Armin Bollow, Thomas Reichelt, Dominik Ellguth, Niko Engelke, Dirk Große Meininghaus, Muharrem Akin, Thomas Bertsch, Ibrahim Akin, Michael Behnes

**Affiliations:** 1 Department of Cardiology, Angiology, Haemostaseology and Medical Intensive Care, University Medical Centre Mannheim, Medical Faculty Mannheim, Heidelberg University, Heidelberg, Germany; 2 European Center for AngioScience (ECAS) and German Center for Cardiovascular Research (DZHK) partner site Heidelberg/Mannheim, Mannheim, Germany; 3 Department of Nuclear Medicine, University Hospital Heidelberg, Heidelberg, Germany; 4 Institute of Clinical Chemistry and Laboratory Medicine, University Medical Center Mannheim, University of Heidelberg, Heidelberg, Germany; 5 Department of Cardiology, Carl-Thiem-Klinikum Cottbus, Cottbus, Germany; 6 Department of Cardiology and Angiology, Hannover Medical School, Hannover, Germany; 7 Institute of Clinical Chemistry, Laboratory Medicine and Transfusion Medicine, Nuremberg General Hospital, Paracelsus Medical University, Nuremberg, Germany

**Keywords:** Age, Ventricular arrhythmia, Cardiac arrest, Mortality, Risk stratification, Alter, Mortalität, Ventrikuläre Rhythmusstörungen, Herzstillstand, Risikostratifizierung

## Abstract

**Background:**

This study evaluated the prognostic impact of age on patients presenting with ventricular tachyarrhythmias (VTA) and aborted cardiac arrest.

**Material and methods:**

The present registry-based, monocentric cohort study included all consecutive patients presenting at the University Medical Center Mannheim (UMM) between 2002 and 2016 with ventricular tachycardia (VT), ventricular fibrillation (VF) and aborted cardiac arrest. Middle-aged (40–60 years old) were compared to older patients (> 60 years old). Furthermore, age was analyzed as a continuous variable. The primary endpoint was all-cause mortality at 2.5 years. The secondary endpoints were cardiac death at 24 h, all-cause mortality at index hospitalization, all-cause mortality after index hospitalization and the composite endpoint at 2.5 years of cardiac death at 24 h, recurrent VTA, and appropriate implantable cardioverter defibrillator (ICD) treatment.

**Results:**

A total of 2259 consecutive patients were included (28% middle-aged, 72% older). Older patients were more often associated with all-cause mortality at 2.5 years (27% vs. 50%; hazard ratio, HR = 2.137; 95% confidence interval, CI 1.809–2.523, *p* = 0.001) and the secondary endpoints. Even patient age as a continuous variable was independently associated with mortality at 2.5 years in all types of VTA. Adverse prognosis in older patients was demonstrated by multivariate Cox regression analyses and propensity score matching. Chronic kidney disease (CKD), systolic left ventricular dysfunction (LVEF) < 35%, cardiopulmonary resuscitation (CPR) and cardiogenic shock worsened the prognosis for both age groups, whereas acute myocardial infarction (STEMI/NSTEMI) and the presence of an ICD improved prognosis**.**

**Conclusion:**

The results of this study suggest that increasing age is associated with increased mortality in VTA patients. Compared to the middle-aged, older patients were associated with higher all-cause mortality at 2.5 years and the secondary endpoints.

**Supplementary Information:**

The online version of this article (10.1007/s00391-022-02131-6) contains supplementary material, which is available to authorized users.

## Abstract (de)

**Hintergrund:**

In dieser Studie wurde der prognostische Einfluss des Alters bei Patienten mit ventrikulärer Tachyarrhythmie (VTA) und überlebtem Herzstillstand untersucht.

**Material und Methoden:**

Die vorliegende registerbasierte, monozentrische Kohortenstudie umfasste alle konsekutiven Patienten, die zwischen 2002 und 2016 mit ventrikulärer Tachykardie (VT), Kammerflimmern (VF) und überlebtem Herzstillstand in der Universitätsmedizin Mannheim (UMM) vorgestellt wurden. Patienten mittleren Alters (40–60 Jahre) wurden mit älteren Patienten (> 60 Jahre) verglichen. Außerdem wurde das Alter als kontinuierliche Variable analysiert. Der primäre Endpunkt war die Gesamtmortalität nach 2,5 Jahren. Die sekundären Endpunkte waren der Herztod innerhalb von 24 h, die Gesamtmortalität bei der Indexeinweisung, die Gesamtmortalität nach der Indexeinweisung und der zusammengesetzte Endpunkt nach 2,5 Jahren bestehend aus: Herztod nach 24 h, rezidivierende VTA und angemessene Behandlung mit einem implantierbaren Kardioverter-Defibrillator (ICD).

**Ergebnisse:**

Insgesamt wurden 2259 konsekutive Patienten eingeschlossen (28 % mittleren Alters, 72 % älter). Ältere Patienten waren häufiger mit der Gesamtmortalität nach 2,5 Jahren (27 % vs. 50 %; Hazard-Ratio [HR] = 2,137; 95 % Konfidenzintervall [KI] 1,809–2,523; *p* = 0,001) und den sekundären Endpunkten assoziiert. Selbst das Alter der Patienten als kontinuierliche Variable war bei allen Arten von VTA unabhängig mit der Sterblichkeit nach 2,5 Jahren verbunden. Eine ungünstige Prognose bei älteren Patienten wurde durch multivariate Cox-Regressionsanalysen und Propensity-Score-Matching nachgewiesen. Chronische Nierenerkrankung (CKD), hochgradig reduzierte systolische linksventrikuläre Funktion (LVEF) < 35 %, kardiopulmonale Reanimation (CPR) und kardiogener Schock verschlechterten die Prognose für beide Altersgruppen, während ein akuter Myokardinfarkt (STEMI/NSTEMI) und das Vorhandensein eines ICD die Prognose verbesserten.

**Schlussfolgerung:**

Die Ergebnisse dieser Studie deuten darauf hin, dass zunehmendes Alter mit einer erhöhten Sterblichkeit bei VTA-Patienten verbunden ist. Im Vergleich zu Patienten mittleren Alters waren ältere Patienten mit einer höheren Gesamtmortalität nach 2,5 Jahren und den sekundären Endpunkten assoziiert.

## Introduction

Age is the main risk factor for vascular disease and therefore for cardiovascular and cerebrovascular events [1]. The incidence of VTA and cardiac death increases with age [2, 3]. VTA in geriatric patients is caused mostly by the increased prevalence of structural heart diseases as a consequence of arterial hypertension, coronary artery disease (CAD), and heart failure [4]. Data regarding the long-term mortality of geriatric patients with ventricular tachyarrhythmias is rare [4, 5]. Therefore, the present study investigated the clinical characteristics of older (> 60 years old) and middle-aged (40–60 years old) patients and evaluates the prognostic impact of age compared to other clinical parameters on the short-term and long-term outcomes of patients presenting with VTA and aborted cardiac arrest on hospital admission.

## Methods

### Study patients, design, and data collection

The present study is derived from an analysis of the Registry of Malignant Arrhythmias and Sudden Cardiac Death—Influence of Diagnostics and Interventions (RACE-IT) and presents a single-center registry of consecutive patients presenting to the UMM between 2002 and 2016 with VTA and SCD (clinicaltrials.gov identifier: NCT02982473; date of registration 5 December 2016) as previously published [6, 7] (suppl. Fig. 1, flowchart). The registry was established according to the principles of the Declaration of Helsinki and was approved by the Ethics Committee II of the Faculty of Medicine Mannheim, University of Heidelberg, Germany. VTA was defined according to current guidelines as previously published [2, 7].

### Definition of study groups and inclusion and exclusion criteria

Risk in the present analysis was stratified according to age, with both age as a binary and age as a continuous variable. For analysis with age as a binary variable, middle-aged (40–60 years old) patients were compared to older patients (> 60 years old) [8]. Furthermore, patients > 75 years were compared to patients < 75 years. Patients younger than 40 years old were excluded.

### Study endpoints

The primary endpoint was all-cause mortality at long-term follow-up of 2.5 years. The secondary endpoints were cardiac death at 24 h, all-cause mortality at index hospitalization, all-cause mortality after index hospitalization and the composite endpoint at 2.5 years of cardiac death at 24 h, recurrent VTA and appropriate ICD treatment.

As previously published, the statistical methods included multivariate Cox regression models, Kaplan-Meier analyses, and propensity score matching [7].

## Results

### Study population before propensity score matching

The present study included a total of 2259 consecutive patients presenting with VTA and aborted cardiac arrest. Of these, 28% were middle-aged (40–60 years old) and 72% were older (> 60 years old) (suppl. Fig. 1, flowchart). As outlined in Table 1, older patients showed higher rates of VT and middle-aged patients showed higher rates of VF. Middle-aged patients had higher rates of CPR due mainly to out-of-hospital CPR. Older patients suffered more often from arterial hypertension, diabetes mellitus and hyperlipidemia, whereas middle-aged patients showed higher rates of a family history of cardiac diseases and smoking. Prior CAD and prior myocardial infarction were more common in older patients; however, middle-aged patients had higher rates of acute myocardial infarction, coronary angiography and electrophysiological examination at index. Nonischemic cardiomyopathy was more frequent among the middle-aged and atrial fibrillation was more frequent among the older patients, besides CKD and chronic obstructive pulmonary disease (COPD). Older patients showed higher rates of prior heart failure, acute heart failure at index and highly restricted LVEF < 35%. Furthermore, older patients had higher rates of device treatment and they were more likely to take beta blockers, ACE inhibitors, angiotensin receptor blockers, statins, amiodarone and digitalis. Study population after propensity score matching is shown in suppl. Table 4.

**Table 1:** Baseline characteristics before propensity matching

Characteristic	40–60 years old (*n* = 628; 28%)		> 60 years old (*n* = 1631; 72%)		*p* value
**Age**, median years (range)	52 (40–60)		73 (61–97)		**0.001**
**Male gender**, *n* (%)	456	(73)	1184	(73)	0.993
**Ventricular tachyarrhythmias at index**, *n* (%)					
*VT*	311	(50)	960	(59)	**0.001**
*Sustained*	138	(44)	437	(46)	0.724
*Non-sustained*	173	(28)	523	(32)	**0.037**
*Induced*	110	(37)	282	(30)	**0.034**
Fast	292	(98)	916	(99)	0.547
Slow	6	(2)	14	(2)	
Monomorphic	290	(97)	898	(97)	0.522
Polymorphic	8	(3)	32	(3)	
*VF*	317	(50)	671	(41)	**0.001**
**Cardiopulmonary resuscitation**, *n* (%)	314	(50)	772	(47)	**0.001**
*In-hospital*	94	(15)	365	(22)	
*Out-of-hospital*	220	(35)	407	(25)	
**Cardiovascular risk factors, ***n* (%)					
*Arterial hypertension*	286	(46)	1062	(65)	**0.001**
*Diabetes mellitus*	102	(16)	526	(32)	**0.001**
*Hyperlipidemia*	157	(25)	492	(30)	**0.015**
*Smoking*	262	(42)	352	(22)	**0.001**
*Cardiac family history*	94	(15)	102	(6)	**0.001**
**Comorbidities, ***n* (%)					
*Prior myocardial infarction*	97	(15)	446	(27)	**0.001**
*Prior coronary artery disease*	154	(25)	790	(48)	**0.001**
*Prior heart failure*	105	(17)	443	(27)	**0.001**
*Prior PTCA*	91	(15)	398	(24)	**0.001**
*Prior CABG*	35	(6)	263	(16)	**0.001**
*Atrial fibrillation*	95	(15)	612	(38)	**0.001**
Paroxysmal	77	(12)	421	(26)	**0.001**
Persisting	12	(2)	48	(3)	
Permanent	6	(1)	143	(9)	
*Nonischemic cardiomyopathy*	47	(8)	78	(5)	**0.012**
*Chronic kidney disease*	231	(38)	928	(59)	**0.001**
*COPD*	27	(4)	180	(11)	**0.001**
*Asthma*	5	(1)	16	(1)	0.682
**Comorbidities at index, ***n* (%)					
*Cardiogenic shock*	46	(7)	236	(15)	**0.001**
*Acute heart failure*	90	(14)	298	(18)	**0.026**
**Acute myocardial infarction at index**, *n* (%)	217	(35)	459	(28)	**0.003**
*STEMI*	93	(15)	140	(9)	**0.001**
*NSTEMI*	124	(20)	319	(20)	0.920
**Coronary angiography at index**, *n* (%)	409	(65)	960	(59)	**0.006**
*No evidence of CAD*	122	(30)	213	(22)	**0.001**
*1‑vessel disease*	117	(29)	195	(20)	
*2‑vessel disease*	97	(24)	237	(25)	
*3‑vessel disease*	73	(18)	315	(33)	
*Presence of chronic total occlusion*	55	(13)	235	(25)	0.001
*Presence of CABG*	28	(7)	155	(16)	0.001
*PCI*	217	(53)	415	(43)	**0.001**
**Left ventricular ejection fraction, ***n* (%)					
*Not documented*	171	(27)	433	(27)	**1.000**
*>* *55%*	170	(27)	297	(18)	**0.001**
*45–54%*	65	(10)	161	(10)	
*35–44%*	85	(14)	248	(15)	
*<* *35%*	137	(22)	492	(30)	
**Cardiac treatment at index**, *n* (%)					
*Electrophysiological examination*	177	(28)	340	(21)	**0.001**
*VT ablation treatment*	36	(6)	70	(4)	0.147
**Device treatment overall, ***n* (%)	226	(46)	555	(52)	**0.016**
**Medication at discharge**, *n* (%)					
*Beta blocker*	390	(79)	891	(84)	**0.016**
*ACE inhibitor*	304	(61)	713	(67)	**0.027**
*ARB*	39	(8)	145	(14)	**0.001**
*Statin*	301	(61)	704	(66)	**0.035**
*Amiodarone*	54	(11)	200	(19)	**0.001**
*Digitalis*	43	(9)	163	(15)	**0.001**
*Aldosterone antagonist*	54	(11)	121	(11)	0.781
**Follow-up times**					
*Hospitalization total, days- median (IQR)*	10	(4–17)	11	(5–22)	**0.001**
*ICU time, days- median (IQR)*	2	(0–7)	3	(0–8)	0.134
*Follow-up, days- mean; median (range)*	1832; 1709		1116; 491		**0.001**
	(0–5089)		(0–5106)		

Bold type indicates statistical significance *p* < 0.05

*ACE* angiotensin-converting enzyme, *ARB* angiotensin receptor blocker, *CABG* coronary artery bypass grafting, *CAD* coronary artery disease, *COPD* chronic obstructive pulmonary disease, *ICU* intensive care unit, *IQR* interquartile range, *NSTEMI* non-ST-segment myocardial infarction, *PCI* percutaneous coronary intervention, *PTCA* percutaneous transluminal coronary angioplasty, *STEMI* ST-segment myocardial infarction, *VF* ventricular fibrillation, *VT* ventricular tachycardia

### Primary and secondary endpoints before propensity score matching

As shown in Table 2, left panel, older patients > 60 years old in the unmatched cohort showed higher rates of all-cause mortality at 2.5 years (27% vs. 50%, *p* = 0.001, hazard ratio, HR = 2.137, 95% confidence interval, CI 1.809–2.523, *p* = 0.001), cardiac death at 24 h and all-cause mortality at index hospitalization and after index hospitalization. Furthermore, older patients showed higher rates of the composite endpoint at 2.5 years (24% vs. 34%, *p* = 0.001, HR = 1.471; 95% CI 1.230–1.759, *p* = 0.001). Even after propensity score matching, older patients showed increased mortality at 2.5 years, as shown in Table 2, right panel.

**Table 2:** Primary and secondary endpoints for all patients before and after propensity score matching

	Before matching					After matching				
Characteristic	40–60 years old (*n* = 628; 28%)		> 60 years old (*n* = 1631; 72%)		*p* value	40–60 years old (*n* = 442; 50%)		> 60 years old (*n* = 442; 50%)		*p* value
*Primary endpoint, n (%)*										
All-cause mortality at 2.5 years	168	(27)	812	(50)	**0.001**	83	(19)	156	(35)	**0.001**
*Secondary endpoints, n (%)*										
Cardiac death at 24 h	80	(13)	335	(21)	**0.001**	25	(6)	40	(9)	0.053
All-cause mortality at index hospitalization	131	(21)	564	(35)	**0.001**	57	(13)	86	(20)	**0.001**
All-cause mortality after index hospitalization	96	(15)	536	(33)	**0.001**	81	(18)	154	(35)	**0.001**
Composite endpoint at 2.5 years (Cardiac death at 24 h, recurrent ventricular tachyarrhythmias, appropriate ICD treatment)	153	(24)	557	(34)	**0.001**	80	(18)	109	(25)	**0.020**

*ICD* implantable cardioverter-defibrillator

### Multivariate Cox regression models before propensity score matching with age as binary variable

Age was significantly associated with the primary endpoint all-cause mortality at 2.5 years. Other predictors of this endpoint were CKD, LVEF < 35%, cardiogenic shock, CPR and male gender. The presence of an ICD and AMI were beneficial. (Table 3, upper panel). Age > 60 years was also significantly associated with the composite endpoint at 2.5 years. Other predictors of this endpoint were cardiogenic shock, ICD, LVEF < 35%, CPR and CKD; however, STEMI was not significantly associated with this endpoint (Table 3, lower panel). Furthermore, patient age as a continuous variable was independently associated with mortality at 2.5 years in all types of VTA (suppl. Tables 1–3).

**Table 3:** Multivariate Cox regression analyses for all patients before propensity score matching

Endpoint	HR	95% CI	*p* value
*All-cause mortality at 2.5 years*			
Male gender	1.283	1.059–1.554	**0.011**
Chronic kidney disease	2.453	2.022–2.976	**0.001**
Diabetes	1.090	0.914–1.300	0.336
STEMI	0.504	0.362–0.703	**0.001**
NSTEMI	0.787	0.634–0.975	**0.029**
Cardiogenic shock	1.796	1.479–2.182	**0.001**
CPR	1.745	1.566–1.944	**0.001**
ICD	0.239	0.193–0.296	**0.001**
LVEF < 35%	2.039	1.713–2.427	**0.001**
CAD	0.879	0.722–1.070	0.200
Age > 60 years	2.068	1.627–2.630	**0.001**
*Composite endpoint at 2.5 years*			
Male gender	1.002	0.802–1.250	0.989
Chronic kidney disease	1.355	1.105–1.661	**0.004**
Diabetes	0.905	0.733–1.118	0.356
STEMI	0.456	0.282–0.738	**0.001**
NSTEMI	0.879	0.671–1.151	0.348
Cardiogenic shock	1.502	1.167–1.933	**0.002**
CPR	1.371	1.202–1.564	**0.001**
ICD	1.451	1.173–1.794	**0.001**
LVEF < 35%	1.437	1.175–1.758	**0.001**
CAD	0.844	0.676–1.055	0.136
Age > 60 years	1.621	1.261–2.084	**0.001**

Bold type indicates statistical significance *p* < 0.05

*CAD* coronary artery disease, *CI* confidence interval, *HR* hazard ratio, *CPR* cardiopulmonary resuscitation, *ICD* implantable cardioverter-defibrillator, *LVEF* left ventricular ejection fraction, *NSTEMI* non-ST-segment myocardial infarction, *STEMI* ST-segment myocardial infarction

### Kaplan-Meier analyses after propensity score matching

**Fig. 1:**
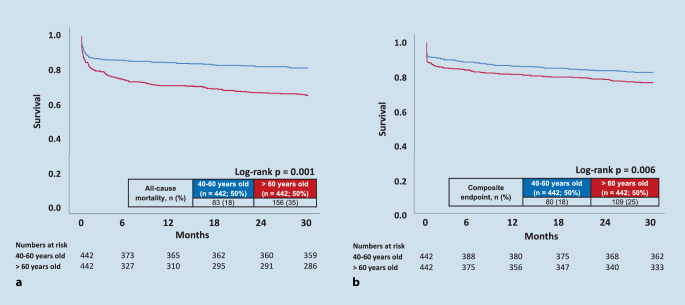
Older patients > 60 years old presenting with ventricular tachyarrhythmias and aborted cardiac arrest were associated with all-causemortality at 2.5 years (**a**) and with the composite endpoint at 2.5 years of cardiac death at 24 h, recurrent ventricular tachyarrhythmias and appropriate ICD treatment (**b**)

As shown in Fig. 1, older patients > 60 years had a worse long-term prognosis for all-cause mortality (18% vs. 35%, *p* = 0.001, HR = 2.023; 95% CI 1.550–2.641, *p* = 0.001) and the composite endpoint at 2.5 years (18% vs. 25%, *p* = 0.006, HR = 1.401; 95% CI 1.050–1.870, *p* = 0.020). Furthermore, patients ≥ 75 years were associated with increased mortality at 2.5 years and an increased risk of the composite endpoint (suppl. Fig. 2).

### Multivariate Cox regression analysis

The multivariate Cox regression model values in Table 4 show consistent significant associations of CKD, LVEF < 35% and CPR with all-cause mortality at 2.5 years and the composite endpoint at 2.5 years for both middle-aged (40–60 years old) and older patients > 60 years old. In contrast, STEMI and ICD at index were beneficial. There was an association among middle-aged patients after cardiogenic shock and ICD treatment with all-cause mortality at 2.5 years, despite a lack of association with the composite endpoint.

**Table 4:** Multivariate Cox regression models for middle-aged (40–60 years old) and older patients > 60 years old

		All-cause mortality at 2.5 years					Composite endpoint at 2.5 years (Cardiac death at 24 h, recurrent ventricular tachyarrhythmias, appropriate ICD treatment)					
Variables	40–60 years			> 60 years			40–60 years			> 60 years		
	HR	95% CI	*p* value	HR	95% CI	*p* value	HR	95% CI	*p* value	HR	95% CI	*p* value
Male gender	1.430	0.864–2.365	0.164	1.263	1.025–1.556	**0.028**	0.981	0.593–1.621	0.939	1.013	0.790–1.298	0.921
Diabetes	1.211	0.694–2.113	0.501	1.135	0.941–1.369	0.185	0.494	0.241–1.013	0.054	0.950	0.758–1.190	0.656
Chronic kidney disease	3.226	1.939–5.365	**0.001**	2.261	1.833–2.789	**0.001**	1.751	1.106–2.774	**0.017**	1.292	1.028–1.622	**0.027**
Atrial fibrillation	1.398	0.805–2.427	0.234	1.101	0.919–1.319	0.295	1.598	0.958–2.664	0.072	0.831	0.668–1.033	0.096
Cardiogenic shock	1.929	1.148–3.240	**0.001**	1.755	1.421–2.168	**0.001**	1.398	0.705–2.773	0.337	1.525	1.158–2.010	**0.003**
CPR	2.031	1.439–2.866	**0.001**	1.663	1.455–1.901	**0.001**	1.607	1.117–2.311	**0.011**	1.470	1.245–1.735	**0.001**
Coronary artery disease	0.781	0.456–1.338	0.368	0.940	0.757–1.167	0.577	0.609	0.363–1.021	0.060	0.919	0.712–1.185	0.517
NSTEMI	0.427	0.205–0.890	**0.023**	0.875	0.696–1.100	0.253	0.659	0.296–1.468	0.307	0.988	0.739–1.323	0.938
STEMI	0.395	0.175–0.883	**0.024**	0.523	0.360–0.762	**0.001**	0.747	0.286–1.953	0.552	0.421	0.237–0.746	**0.003**
ICD	0.118	0.061–0.227	**0.001**	0.260	0.207–0.327	**0.001**	1.424	0.857–2.364	0.172	1.494	1.180–1.893	**0.001**
LVEF < 35%	2.303	1.429–3.712	**0.001**	1.902	1.575–2.297	**0.001**	1.654	1.028–2.661	**0.038**	1.378	1.103–1.723	**0.005**
Ventricular fibrillation	0.828	0.459–1.494	0.531	1.091	0.871–1.342	0.478	0.455	0.254–0.816	0.008	0.868	0.666–1.130	0.293

Bold type indicates statistical significance *p* < 0.05

*CPR* cardiopulmonary resuscitation, *ICD* implantable cardioverter-defibrillator, *LVEF* left ventricular ejection fraction, *NSTEMI* non-ST-segment elevation myocardial infarction, *STEMI* ST-segment elevation myocardial

## Discussion

The results of the present study suggest that increasing age is associated with increased mortality in VTA patients. Compared to the middle-aged (40–60 years old), older patients > 60 years old were associated with higher all-cause mortality at 2.5 years, all-cause mortality at index hospitalization and after index hospitalization, cardiac death at 24 h, and the composite endpoint at 2.5 years. The overall all-cause mortality rate in Germany in 2015 was far lower than that seen is this study; however, the mortality rate for the elderly was 12 times higher than that of the middle-aged (0.97% ÷ 0.08% = 12) (© Statistisches Bundesamt [Destatis], 2021).

Compared to the general population the overall 2015 all-cause mortality rate within our university medical centre across all fields of specializations was higher than in the general population but for patients > 60 years old only 2.8 times higher than that of middle-aged patients (4.8% ÷ 1.7% = 2.8).

This increase in mortality rate is related to the disease severity and the number of affected patients in the hospital population; however, the lower ratio of age-dependent mortality rates in the hospital population is caused by the preselection of diseased people and the exclusion of healthy individuals, who are more numerous in the general population. Respectively within a cardiologic department. Here, the mortality rate for elderly patients is only 1.6 times higher than that of middle-aged patients (10.3% ÷ 6.3% = 1.6). In this context, all-cause mortality rates in the present preselected cohort of patients with VTA are even higher, whereas the ratio between age groups is further reduced (before propensity score matching: 50% ÷ 27% = 1.85; after propensity score matching: 34% ÷ 18% = 1.9).

In daily clinical routine patients age is regarded as one of the highest prognosis-limiting factors and geriatric patients are predicted to have the worst prognosis. The present data suggests that patient age influences mortality in VTA patients but has less influence on mortality then in the general and the overall hospital population. Therefore, risk stratification in VTA patients should not be applied only by chronological age and needs to be seen in context with other comorbidities that influence the biological age of a patient.

The biological vascular age is determined by chronic diseases, such as CDK and heart failure that are in a bidirectional relationship with functional and structural changes in vessels, such as arterial wall stiffness, arterial hypertension, intima thickening and endothelial dysfunction [1].

The present study revealed that CKD and heart failure with LVEF < 35% on admission are consistently associated with an adverse prognosis for mortality, cardiac death, and recurrent VTA for both middle-aged and older patients. This suggests that besides the chronological age the biological age influences mortality in VTA patients.

In the present study a beneficial effect of an ICD on the prevention of all-cause mortality at 2.5 years, cardiac death at 24 h, recurrent ventricular tachyarrhythmias, and ICD treatment in patients > 60 years old was shown. In general, the ICD implantation effectively decreased long-term mortality in patients with LVEF < 35% irrespective of the underlying type of heart failure. International guidelines recommend implanting an ICD at any age when assuming a life expectancy of at least 1 year [2, 9]; however, clinical trials on ICDs frequently exclude geriatric patients [4, 10, 11], which raises doubts about the benefit, efficacy and safety of ICD implantation in geriatric patients [2]. Therefore, further studies on geriatric patients examining the safety and effectiveness of the ICD would be desirable.

There is no distinctive guideline-recommended treatment for geriatric patients presenting with ventricular tachyarrhythmias, such as specific antiarrhythmic drug treatment and VT catheter ablation, because older patients frequently suffer from various heterogeneous comorbidities [10]. Furthermore, older patients commonly suffer adverse side effects from antiarrhythmic drugs because of decreased physiological function, polypharmacy, and frailty syndrome [10, 12]. Therefore, geriatric patients in particular should receive individualized treatment designed by multidisciplinary teams, as they are in greater danger of ventricular tachyarrhythmias and sudden cardiac arrest.

## Study limitations

Study limitations were previously published [7]. The ICD programming changed during the last years, mainly due to the knowledge of the MADIT-RIT study (Multicenter Automatic Defibrillator Implantation Trial–Reduce Inappropriate Therapy) in 2012 and might have influenced the endpoints in the present study [13]. Due to the study’s retrospective nature, no geriatric assessments were carried out and documented, which could be included in the evaluation of a patient’s prognosis.

## Conclusion

The results of the present study suggest that increasing age is associated with increased mortality in VTA patients. Compared to the middle-aged (40–60 years old), older patients > 60 years old were associated with higher all-cause mortality at 2.5 years, all-cause mortality at index hospitalization and after index hospitalization, cardiac death at 24 h, and the composite endpoint at 2.5 years. In both middle-aged and older patients CKD and LVEF < 35% were associated with impaired prognosis at 2.5 years, which implies a high impact of both chronological and biological age on mortality of VTA patients. The presence of an ICD predicted better prognosis in both middle-aged and older patients.

### Supplementary Information

Suppl. Fig. 1: Flow chart of selection of 2422 consecutive patients presenting between 2002 and 2016 with ventricular tachyarrhythmias and aborted cardiac arrest on admission.

Suppl. Fig. 2: Patients ≥ 75 years presenting with ventricular tachyarrhythmias were associated with all-cause mortality at 2.5 years (left panel) and with the composite endpoint at 2.5 years of cardiac death at 24 h, recurrent ventricular tachyarrhythmias and appropriate ICD treatment (right panel).

Suppl. Tab. 1. Multivariable Cox regression analyses for patients presenting with ventricular fibrillation

Suppl. Tab. 2. Multivariable Cox regression analyses for patients presenting with sustained ventricular tachycardia

Suppl. Tab. 3. Multivariable Cox regression analyses for patients presenting with non-sustained ventricular tachycardia

Suppl. Tab. 4. Baseline characteristics after propensity score matching

## Abbreviations

CAD: Coronary artery disease
CKD: Chronic kidney disease
CPR: Cardiopulmonary resuscitation
ICD: Implantable cardioverter defibrillator
LVEF: Left ventricular ejection fraction
NSTEMI: Non-ST-segment elevation myocardial infarction
NSVT: Non-sustained ventricular tachycardia
STEMI: ST-segment elevation myocardial infarction
SVT: Sustained ventricular tachycardia
VF: Ventricular fibrillation
VT: Ventricular tachycardia
VTA: Ventricular tachyarrhythmia

## Declarations

For this article no studies with human participants or animals were performed by any of the authors. All studies mentioned were in accordance with the ethical standards indicated in each case.
