# Supplementary material for: Impact of age on the prognosis of patients with ventricular tachyarrhythmias and aborted cardiac arrest
Source: Z Gerontol Geriatr. 2022 Dec 8;56(6):484–91. doi: 10.1007/s00391-022-02131-6 (PMC10522500; doi:10.1007/s00391-022-02131-6)
Supplement: Supplementary file 1 — Suppl. Fig. 1: Flow chart of selection of 2422 consecutive patients presenting between 2002 and 2016 with ventricular tachyarrhythmias and aborted cardiac arrest on admission. [file 391_2022_2131_MOESM1_ESM.pdf]

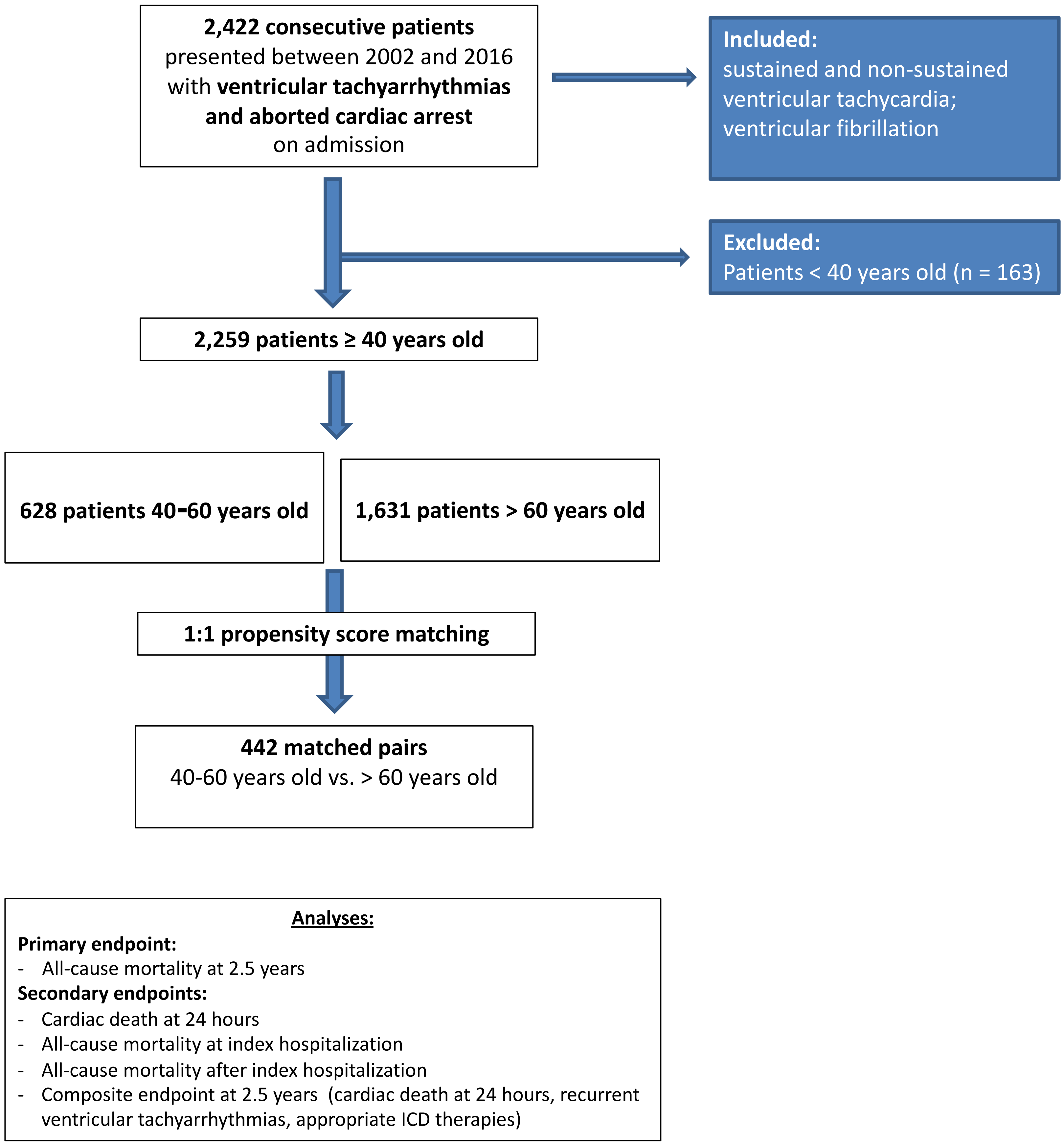
2,422 consecutive patients presented between 2002 and 2016 with ventricular tachyarrhythmias and aborted cardiac arrest on admission
Included: sustained and non-sustained ventricular tachycardia; ventricular fibrillation
Excluded: Patients < 40 years old (n = 163)
2,259 patients ≥ 40 years old
628 patients 40-60 years old
1,631 patients > 60 years old
1:1 propensity score matching
442 matched pairs 40-60 years old vs. > 60 years old
Analyses:
Primary endpoint:
- All-cause mortality at 2.5 years
Secondary endpoints:
- Cardiac death at 24 hours
- All-cause mortality at index hospitalization
- All-cause mortality after index hospitalization
- Composite endpoint at 2.5 years (cardiac death at 24 hours, recurrent ventricular tachyarrhythmias, appropriate ICD therapies)
