# Supplementary figures and images for: Impact of age on the prognosis of patients with ventricular tachyarrhythmias and aborted cardiac arrest
Source: Z Gerontol Geriatr. 2022 Dec 8;56(6):484–91. doi: 10.1007/s00391-022-02131-6 (PMC10522500; doi:10.1007/s00391-022-02131-6)

## after propensity score matching

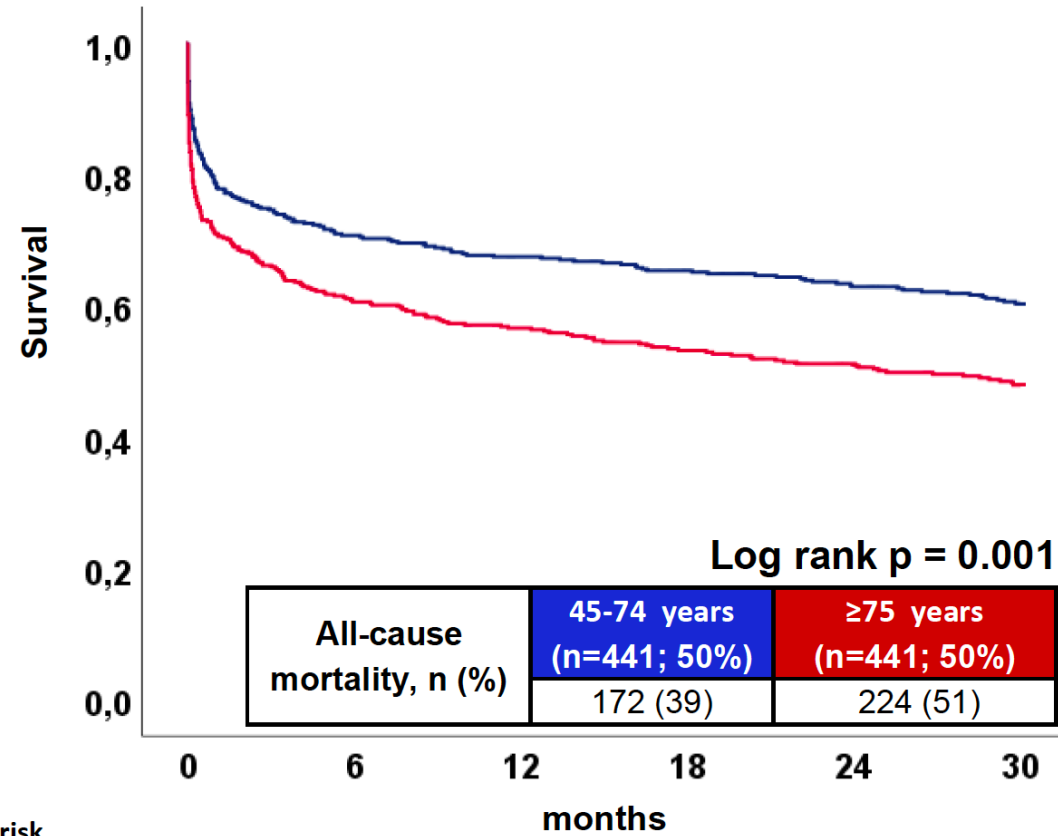

Numbers at risk

|           |     |     |     |     |     |     |
|-----------|-----|-----|-----|-----|-----|-----|
| <75 years | 441 | 307 | 289 | 273 | 252 | 236 |
| ≥75 years | 441 | 260 | 237 | 213 | 195 | 175 |

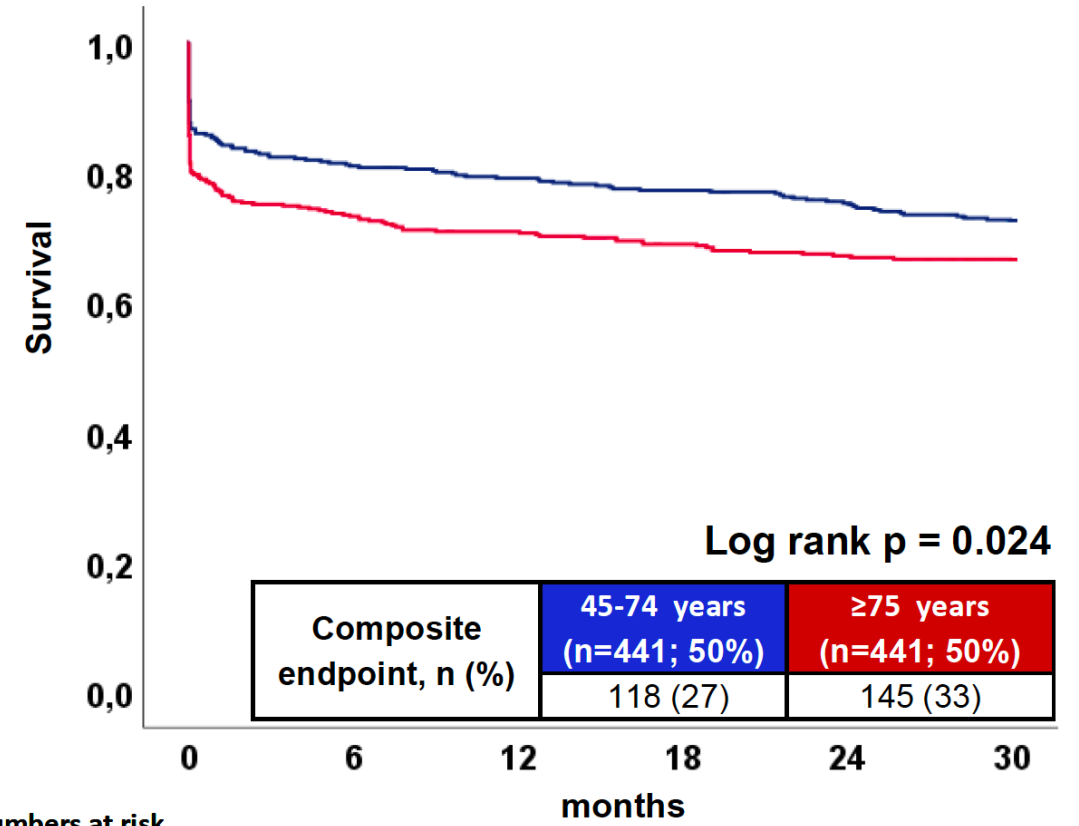

Numbers at risk

|           |     |     |     |     |     |     |
|-----------|-----|-----|-----|-----|-----|-----|
| <75 years | 441 | 351 | 338 | 321 | 299 | 281 |
| ≥75 years | 441 | 314 | 299 | 276 | 254 | 237 |

Supplement: Supplementary file 2 — Suppl. Fig. 2: Patients ≥ 75 years presenting with ventricular tachyarrhythmias were associated with all-cause mortality at 2.5 years (left panel) and with the composite endpoint at 2.5 years of cardiac death at 24 h, recurrent ventricular tachyarrhythmias and appropriate ICD treatment (right panel). [file 391_2022_2131_MOESM2_ESM.pdf]
