# Supplementary material for: Impact of age on the prognosis of patients with ventricular tachyarrhythmias and aborted cardiac arrest
Source: Z Gerontol Geriatr. 2022 Dec 8;56(6):484–91. doi: 10.1007/s00391-022-02131-6 (PMC10522500; doi:10.1007/s00391-022-02131-6)
Supplement: Supplementary file 3 — Suppl. Tab. 1. Multivariable Cox regression analyses for patients presenting with ventricular fibrillation [file 391_2022_2131_MOESM3_ESM.docx]

| **Suppl. Table 1. Multivariable Cox regression analyses for patients presenting with ventricular fibrillation** | | | |
| --- | --- | --- | --- |
| **Endpoint** | **HR** | **95% CI** | **p value** |
| **All-cause mortality at 2.5 years** |  |  |  |
| Male gender | 1.189 | 0.923-1.532 | 0.181 |
| Diabetes | 1.011 | 0.747-1.241 | 0.770 |
| Chronic Kidney disease | 2.253 | 1.902-3.347 | **0.001** |
| CPR | 1.272 | 1.048-1.544 | **0.015** |
| CAD | 0.827 | 0.623-1.096 | 0.186 |
| AMI | 0.592 | 0.452-0.776 | **0.001** |
| ICD | 0.166 | 0.166-0-237 | **0.001** |
| LVEF < 35% | 1.914 | 1.505-2.435 | **0.001** |
| Age | 1.028 | 1.019-1.038 | **0.001** |
| **Composite endpoint at 2.5 years** |  |  |  |
| Male gender | 0.967 | 0.701-1.336 | 0.840 |
| Diabetes | 1.096 | 0.789-1.523 | 0.585 |
| Chronic Kidney disease | 1.542 | 1.103-2.157 | 0.011 |
| CPR | 1.129 | 0.893-1.429 | 0.310 |
| CAD | 0.643 | 0.453-0.911 | **0.013** |
| AMI | 0.728 | 0.505-1.050 | 0.089 |
| ICD | 1.133 | 0.809-1.586 | 0.468 |
| LVEF < 35% | 1.238 | 0.903-1.696 | 0.185 |
| Age | 1.024 | 1.012-1.037 | **0.001** |
| AMI, acute myocardial infarction CAD, coronary artery disease; CI; confidence interval; HR; hazard ratio; CPR, cardiopulmonary resuscitation; ICD; implantable cardioverter-defibrillator; LVEF, left ventricular ejection fraction.  Bold type indicates statistical significance p < 0.05. | | | |
