# Supplementary material for: Impact of age on the prognosis of patients with ventricular tachyarrhythmias and aborted cardiac arrest
Source: Z Gerontol Geriatr. 2022 Dec 8;56(6):484–91. doi: 10.1007/s00391-022-02131-6 (PMC10522500; doi:10.1007/s00391-022-02131-6)
Supplement: Supplementary file 4 — Suppl. Tab. 2. Multivariable Cox regression analyses for patients presenting with sustained ventricular tachycardia [file 391_2022_2131_MOESM4_ESM.docx]

| **Suppl. Table 2. Multivariable Cox regression analyses for patients presenting with sustained ventricular tachycardia** | | | |
| --- | --- | --- | --- |
| **Endpoint** | **HR** | **95% CI** | **p value** |
| **All-cause mortality at 2.5 years** |  |  |  |
| Male gender | 1.414 | 0.941-2.124 | 0.095 |
| Diabetes | 1.022 | 0.678-1.363 | 0.852 |
| Chronic Kidney disease | 2.455 | 1.663-3.624 | **0.001** |
| CPR | 2.556 | 2.070-3.156 | **0.001** |
| CAD | 0.986 | 0.672-1.444 | 0.941 |
| AMI | 0.758 | 0.492-1.167 | 0.208 |
| ICD | 0.267 | 0.179-0.397 | **0.001** |
| LVEF < 35% | 1.731 | 1.216-2.463 | **0.002** |
| Age | 1.022 | 1.007-1.037 | **0.003** |
| **Composite endpoint at 2.5 years** |  |  |  |
| Male gender | 0.956 | 0.666-1.374 | 0.809 |
| Diabetes | 1.002 | 0.653-1.312 | 0.665 |
| Chronic Kidney disease | 1.190 | 0.855-1.655 | 0.302 |
| CPR | 1.632 | 1.308-2.037 | **0.001** |
| CAD | 0.815 | 0.572-1.162 | 0.258 |
| AMI | 1.267 | 0.803-2.000 | 0.309 |
| ICD | 1.487 | 1.016-2.177 | **0.041** |
| LVEF < 35% | 1.227 | 0.891-1.690 | 0.211 |
| Age | 1.005 | 0.993-1.017 | 0.437 |
| AMI, acute myocardial infarction; CAD, coronary artery disease; CI; confidence interval; HR; hazard ratio; CPR, cardiopulmonary resuscitation; ICD; implantable cardioverter-defibrillator; LVEF, left ventricular ejection fraction.  Bold type indicates statistical significance p < 0.05. | | | |
