# Supplementary material for: Impact of age on the prognosis of patients with ventricular tachyarrhythmias and aborted cardiac arrest
Source: Z Gerontol Geriatr. 2022 Dec 8;56(6):484–91. doi: 10.1007/s00391-022-02131-6 (PMC10522500; doi:10.1007/s00391-022-02131-6)
Supplement: Supplementary file 5 — Suppl. Tab. 3. Multivariable Cox regression analyses for patients presenting with non-sustained ventricular tachycardia [file 391_2022_2131_MOESM5_ESM.docx]

| **Suppl. Table 3. Multivariable Cox regression analyses for patients presenting with non-sustained ventricular tachycardia** | | | |
| --- | --- | --- | --- |
| **Endpoint** | **HR** | **95% CI** | **p value** |
| **All-cause mortality at 2.5 years** |  |  |  |
| Male gender | 1.928 | 1.234-3.012 | **0.004** |
| Diabetes | 1.891 | 1.329-2.691 | **0.001** |
| Chronic Kidney disease | 2.240 | 1.559-3.218 | **0.001** |
| CPR | 1.686 | 1.284-2.215 | **0.001** |
| CAD | 0.754 | 0.507-1.121 | 0.163 |
| AMI | 1.159 | 0.749-1.793 | 0.509 |
| ICD | 0.317 | 0.210-0.479 | **0.001** |
| LVEF < 35% | 2.476 | 1.705-3.594 | **0.001** |
| Age | 1.048 | 1.030-1.067 | **0.001** |
| **Composite endpoint at 2.5 years** |  |  |  |
| Male gender | 0.549 | 0.532-1.399 | 0.549 |
| Diabetes | 0.647 | 0.427-1.065 | 0.091 |
| Chronic Kidney disease | 1.210 | 0.810-1.809 | 0.352 |
| CPR | 1.734 | 1.235-2.437 | **0.001** |
| CAD | 0.968 | 0.612-1.531 | 0.890 |
| AMI | 0.905 | 0.519-1.576 | 0.890 |
| ICD | 2.270 | 1.453-3.545 | **0.001** |
| LVEF < 35% | 1.580 | 1.035-2.412 | **0.034** |
| Age | 1.009 | 0.992-1.025 | 0.297 |
| AMI, acute myocardial infarction; CAD, coronary artery disease; CI; confidence interval; HR; hazard ratio; CPR, cardiopulmonary resuscitation; ICD; implantable cardioverter-defibrillator; LVEF, left ventricular ejection fraction.  Bold type indicates statistical significance p < 0.05. | | | |
