# Supplementary material for: Impact of age on the prognosis of patients with ventricular tachyarrhythmias and aborted cardiac arrest
Source: Z Gerontol Geriatr. 2022 Dec 8;56(6):484–91. doi: 10.1007/s00391-022-02131-6 (PMC10522500; doi:10.1007/s00391-022-02131-6)
Supplement: Supplementary file 6 — Suppl. Tab. 4. Baseline characteristics after propensity score matching [file 391_2022_2131_MOESM6_ESM.docx]

| **Suppl. Table 4. Baseline characteristics after propensity score matching** | | | | | |
| --- | --- | --- | --- | --- | --- |
| **Characteristic** | **40–60 years old**  (n = 442; 50%) | | **> 60 years old**  (n = 442; 50%) | | **p value** |
| **Age**, median (range) | 52 (40–60) | | 72 (61–94) | | **0.001** |
| **Male gender**, n (%) | 328 | (74) | 323 | (73) | 0.703 |
| **Ventricular tachyarrhythmias at index**, n (%) |  |  |  |  |  |
| VT | 233 | (53) | 230 | (52) | 0.840 |
| Sustained | 104 | (45) | 94 | (41) | 0.413 |
| Non-sustained | 129 | (55) | 136 | (59) | 0.607 |
| Induced | 79 | (35) | 63 | (28) | 0.104 |
| Fast | 218 | (97) | 219 | (98) | 0.760 |
| Slow | 6 | (3) | 5 | (2) |  |
| Monomorph | 218 | (97) | 217 | (97) | 0.778 |
| Polymorph | 6 | (3) | 7 | (3) |  |
| VF | 209 | (47) | 212 | (48) | 0.840 |
| **Cardiopulmonary resuscitation**, n (%) | 200 | (45) | 210 | (48) | 0.500 |
| In-hospital | 136 | (68) | 120 | (57) |  |
| Out-of-hospital | 64 | (32) | 90 | (43) |  |
| **Cardiovascular risk factors**, n (%) |  |  |  |  |  |
| Arterial hypertension | 225 | (51) | 296 | (67) | **0.001** |
| Diabetes mellitus | 73 | (17) | 99 | (22) | **0.027** |
| Hyperlipidemia | 127 | (29) | 140 | (32) | 0.341 |
| Smoking | 206 | (47) | 116 | (26) | **0.001** |
| Cardiac family history | 77 | (17) | 31 | (7) | **0.001** |
| **Comorbidities**, n (%) |  |  |  |  |  |
| Prior myocardial infarction | 82 | (19) | 107 | (24) | **0.040** |
| Prior coronary artery disease | 128 | (29) | 179 | (41) | **0.001** |
| Prior heart failure | 96 | (22) | 129 | (29) | **0.011** |
| Prior PTCA | 77 | (17) | 96 | (22) | 0.107 |
| Prior CABG | 27 | (6) | 56 | (13) | **0.001** |
| Atrial fibrillation | 74 | (17) | 166 | (38) | **0.001** |
| Paroxysmal | 60 | (81) | 114 | (69) |  |
| Persisting | 10 | (14) | 13 | (8) |  |
| Permanent | 4 | (5) | 39 | (23) |  |
| Nonischemic cardiomyopathy | 47 | (11) | 36 | (8) | 0.205 |
| Chronic kidney disease | 159 | (36) | 166 | (38) | 0.625 |
| COPD | 23 | (5) | 43 | (10) | **0.010** |
| Asthma | 2 | (1) | 2 | (1) | 1.000 |
| **Comorbidities at index**, n (%) |  |  |  |  |  |
| Cardiogenic shock | 57 | (13) | 74  65 | (17) | 0.108 |
| Acute heart failure | 41 | (9) |  | (15) | **0.012** |
| **Acute myocardial infarction at index**, n (%) | 156 | (35) | 155 | (35) | 0.944 |
| STEMI | 65 | (42) | 60 | (38) | 0.233 |
| NSTEMI | 91 | (58) | 95 | (62) | 0.109 |
| **Coronary angiography at index**, n (%) | 320 | (72) | 296 | (67) | 0.079 |
| No evidence of CAD | 95 | (30) | 91 | (30) | **0.011** |
| 1-vessel disease | 91 | (28) | 52 | (18) |  |
| 2-vessel disease | 73 | (23) | 80 | (27) |  |
| 3-vessel disease | 61 | (19) | 73 | (25) |  |
| Presence of chronic total occlusion | 48 | (15) | 64 | (22) | **0.033** |
| Presence of CABG | 24 | (8) | 31 | (11) | 0.196 |
| PCI | 162 | (51) | 139 | (47) | 0.363 |
| **Left ventricular ejection fraction**, n (%) | |  |  |  |  |
| > 55% | 164 | (37) | 150 | (34) | 0.755 |
| 45–54% | 63 | (14) | 68 | (15) |  |
| 35–44% | 82 | (19) | 90 | (20) |  |
| < 35% | 133 | (30) | 134 | (30) |  |
| **Cardiac therapies at index**, n (%) |  |  |  |  |  |
| Electrophysiological examination | 130 | (29) | 105 | (24) | 0.057 |
| VT ablation therapy | 25 | (6) | 16 | (4) | 0.150 |
| **Device therapy overall**, n (%) | 149 | (41) | 114 | (38) | 0.389 |
| **Medication at discharge**, n (%) |  |  |  |  |  |
| Not documented | 137 | (31) | 236 | (53) | **0.001** |
| Beta blocker | 259 | (72) | 188 | (63) | **0.013** |
| ACE inhibitor | 200 | (66) | 141 | (69) | 0.499 |
| ARB | 28 | (9) | 30 | (15) | 0.063 |
| Statin | 201 | (66) | 132 | (64) | 0.671 |
| Amiodarone | 25 | (8) | 20 | (10) | 0.554 |
| Digitalis | 22 | (7) | 15 | (7) | 0.977 |
| Aldosterone antagonist | 29 | (10) | 22 | (10) | 0.665 |
| **Follow-up times** |  |  |  |  |  |
| Hospitalization total, days (median (IQR)) | 11 | (7–19) | 13 | (6–24) | **0.001** |
| ICU time, days (median (IQR)) | 3 | (0–7) | 3 | (0–8) | **0.001** |
| Follow-up, days (mean; median (range)) | 2016; 1938 | | 1551; 1221 | | 0.267 |
|  | (0–5089) | | (0–5106) | |  |
| ACE, angiotensin-converting enzyme; ARB, angiotensin receptor blocker; CABG, coronary artery  bypass grafting; CAD, coronary artery disease; COPD, chronic obstructive pulmonary disease; ICU, invasive care unit; IQR, interquartile range; NSTEMI, non-ST-segment myocardial infarction; PCI, percutaneous coronary intervention; PTCA, percutaneous transluminal coronary angioplasty; STEMI, ST-segment myocardial infarction; VF, ventricular fibrillation; VT, ventricular tachycardia.  Bold type indicates statistical significance p < 0.05. | | | | | |
